# Mechanical Properties of Aeolian Sand Concrete Made from Alkali-Treated Aeolian Sand and Zeolite Powder

**DOI:** 10.3390/ma17071537

**Published:** 2024-03-28

**Authors:** Lisi Wei, Zhanquan Yao, Hao Li, Haolong Guo, Yue Li

**Affiliations:** College of Water Conservancy and Civil Engineering, Inner Mongolia Agricultural University, Hohhot 010010, China; 15754859261@163.com (L.W.); hao.li@imau.edu.cn (H.L.); guohl_9610@163.com (H.G.); ndliyue@163.com (Y.L.)

**Keywords:** eco-friendly concrete, aeolian zeolite powder, alkali excitation, mechanical properties, mechanism

## Abstract

The aim of this study is to promote the application of the excited zeolite powder (ZP)with aeolian sand powder (ASP) in the field of aeolian-sand concrete (ASC) production. This study utilises NaOH to treat composite cementitious materials containing aeolian sand and zeolite powders, which were used to replace 50% of the cement in aeolian-sand concrete (ASC). Production of alkali-inspired cement-based windswept concrete(AAZC).The mechanical properties of treated ASC considerably improved, especially when the NaOH dosage was 4% by mass. After curing this sample (denoted as AAZC-4) for 28 d, its compressive strength improved by 17.2%, and its split tensile increased by 16.3%. Potassium feldspar and montmorillonite in zeolite powder and SiO_2_ in the sand were decomposed by OH^−^ and combined with other elements to generate various silicate gels and A-type potassium zeolite crystals inside the concrete. Microscopic examination showed that the gels and crystals intertwined to fill the pores, decreasing (increasing) the percentage of large (small) pores, thus optimising the pore structure. This substantially improved the mechanical properties of ASC. Freeze–thaw salt-intrusion tests showed that the extent of mass loss, degree of damage and loss of compressive strength of AAZC-4 were similar to those of ordinary concrete but were reduced by 36.8%, 19% and 52.1%, respectively, compared with those of ASC. Therefore, AAZC-4 has a sustainable working performance in chloride-ion permeable environments in cold and arid areas.

## 1. Introduction

Concrete, as the most basic building material, is used on a large scale in China’s houses, bridges, dams, rivers and canals; with the gradual acceleration of China’s infrastructure construction, in China’s cement production and cement use is ranked the first in the world, but the cement industry has a low capacity utilisation rate, inefficient production capacity and slow exit, but also emits a large amount of CO_2_ during the production process [1]. As of 2020, China’s CO_2_ emissions in the cement production industry have reached 1.23 billion tons and maintain the trend of continuous increase [2]. However, according to research, low-carbon concrete can reduce the carbon footprint of concrete by 50 per cent [3] and generate profits of $87.333 billion [4]; many scholars are now adopting new types of cement to achieve the effect of reducing carbon emissions [5,6], so the use of new materials to prepare environmentally friendly concrete by substituting ordinary silicate cement has important practical significance for the sustainable development of energy and the realisation of the strategic goal of “carbon peak, carbon neutral”. Therefore, the use of new materials to replace ordinary silicate cement in the preparation of environmentally friendly concrete is of great significance to the sustainable development of energy and the realisation of the strategic goal of “carbon peak and carbon neutral”.

Zeolite is a natural mineral material with huge reserves in China [7], which is cheap and easy to mine, and the zeolite powder (ZP) prepared by grinding it finely has excellent volcanic ash activity [8]. The early volcanic ash activity of zeolite powder is lower than that of mineral admixtures such as silica fume [9,10], and the addition of ZP to cement paste and concrete can accelerate the hydration process of cement, improve the strength and reduce the internal porosity, which is widely used in some large-scale buildings and water conservancy buildings. Sharbatdar, Sadegh et al. [11,12] showed that, although zeolite powder dosage has an excellent micro-aggregate effect and volcanic ash effect, because of its strong water absorption, in the case of fixed water-cement ratio, there will be a large number of zeolite powder particles can not be dissolved, resulting in a reduction in the effective cementitious material, affecting the hydration reaction, which will lead to zeolite powder dosage of more than 20% of the concrete will lead to the mechanical properties and work performance will be with the increase in the dosage of zeolite powder. Showing a downward trend. This also limits the prospect of applying zeolite powder in large mixes of concrete.

Some researchers chose to grind aeolian sand into aeolian-sand powder (ASP), which they used to replace the cement in the preparation of concrete. They did this because the SiO_2_ and Al_2_O_3_ in the ASP can be converted into active sodium silicate and sodium meta aluminate gels, respectively, which, to some extent, offset the loss of properties due to the reduction in the proportion of cement [12]. Li Genfeng et al. [13] found that by grinding the sand into the anemone powder (ASP), which was used to replace cement in the preparation of concrete, the SiO_2_ and Al_2_O_3_ in the ASP could react with Ca(OH)_2_ produced by the hydration of the cement to form a beneficial gel-like substance, which somewhat offset the loss of properties due to the reduction in the proportion of cement. However, the mechanical properties of concrete decreased substantially when the admixture of fenestrated sand powder exceeded 15% [13], which occurred due to the low content of Ca^2+^ and Al^3+^ in the ASP, which greatly reduced the content of C-S-H and C-A-S-H gels in the system and increased the number of internal macropores and connectivity holes. This reduces the compactness of the concrete and greatly reduces its strength. Therefore, the use of large dosages of ZP and ASP alone to replace cement cannot fulfil the conditions for the preparation of eco-friendly concrete.

To improve the mechanical properties of concrete, many researchers [14] have focused on using alkalis to improve the properties of eco-friendly concrete while keeping costs low. Cement-based materials exhibit higher hydration activity when exposed to alkaline environments, which results in the faster development of rigidity and greater resistance to high-temperature acid corrosion. In particular, treatment with NaOH is the most effective one for pozzolanic materials [15,16]. Several studies have explored the mechanical strength of concrete prepared using various percentages of NaOH [17,18]. The resulting metakaolin material had the highest strength and best durability when NaOH was added at 4% by mass. The optimal percentage of added NaOH for low-calcium volcanic ash and for uncalcined volcanic ash containing potassium zeolite was also found to be 4% because it generated hydration substances such as sodium aluminosilicate hydrate (N-A-S-H) gel, which filled the pores and increased the mechanical properties of the concrete [19]. Thus, treatment with NaOH has a substantial positive impact on the mechanical and physical properties of cement-based materials.

The above research indicates that the amount of single powder added after excitation is relatively small. In order to increase the amount of powder added and determine the content of alkali activator, in this paper, the composite cementitious materials were therefore prepared using ZP and ASP in the ratios 5:5 and using NaOH admixtures of 2%, 4% or 6% were used for the alkali treatment. The composite cementitious materials were used both before and after the alkali treatment to replace 50% of the cement to prepare alkali-treated eco-friendly concrete (AAZC). Compressive-strength tests and split-tensile-strength tests were then performed to investigate the effect of the alkali treatment on the mechanical properties of the AAZC. The results of these tests were combined with the results from scanning electron microscopy (SEM) combined with energy-dispersive X-ray spectroscopy (EDS), thermogravimetry (TG) combined with differential scanning calorimetry (DSC), X-ray diffraction (XRD) spectroscopy, Fourier-transform infrared (FTIR) spectroscopy and nuclear magnetic resonance (NMR) tests to determine the mechanism by which the NaOH treatment influences the mechanical properties of the AAZC. Finally, a freeze–thaw salt-intrusion damage test was performed on the optimised NaOH-treated AAZC, ordinary concrete (OC) and untreated AAZC to determine the sustainable working performance of the optimised NaOH-treated AAZC under the chloride ion permeable environment in cold and arid areas.

## 2. Materials and Methods

### 2.1. Materials and Reagents

**Figure 1:**
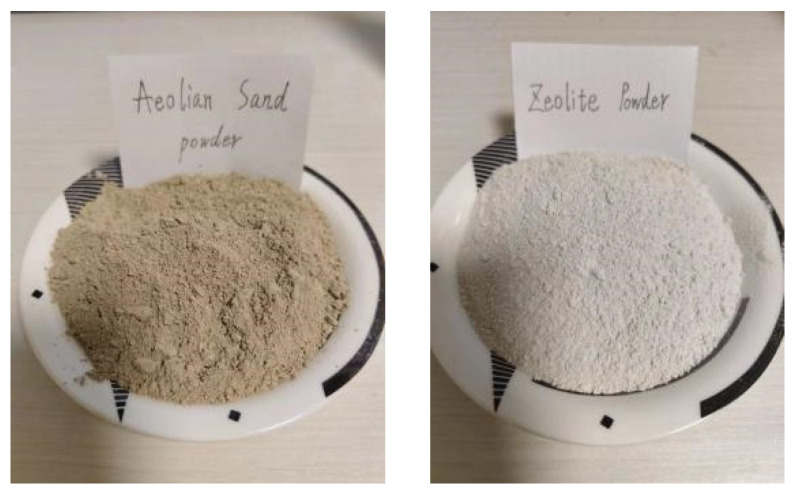
Morphology of airborne sand powder and zeolite powder.

The following materials were used:

- Cement: ordinary Jidong P. O 42.5 silicate cement; its performance characteristics are shown in Table 1.
- Fly ash: class-2 fly ash from the Jinqiao Thermal Power Plant in Hohhot City, Inner Mongolia.
- Coarse aggregate: ordinary crushed-stone pebbles.
- Fine aggregate: Kubuqi Desert aeolian sand and natural river sand.
- Water: ordinary tap water.
- Admixtures: the experimental admixtures used AE-11 high-efficiency air-entraining water-reducing agent produced by Shanxi Qinfen Building Materials Co., Ltd., (Taiyuan, China). Zeolite powder is taken from natural zeolite powder of Lingshou Dehang Mineral Products Co., Ltd., (Shijiazhuang, China). The chemical compositions and particle properties of the aeolian sand powder and the ZP are shown in Table 2. Pictures of ASCP and ZP are shown in Figure 1.

### 2.2. AAZC-4 Concrete Specimens

**Figure 2:**
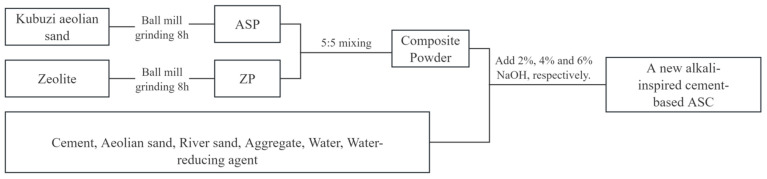
Flowchart of mixing process.

To prepare the eco-friendly concrete (AAZC), according to the ‘Standard concrete mixing performance test method’ (GB/T50080-2016) [20], the ZP and ASP were mixed in the ratios of 5:5 and; these mixtures were used as the composite gel materials to replace 50% of the cement in each concrete sample. The production flowchart is shown in Figure 2.

The abbreviation AAZC-A represents the concrete sample of AAZC prepared using the alkali-treated ZP and ASP, with A indicating the mass ratio of NaOH. The concrete samples obtained using 2%, 4% and 6% NaOH are designated AAZC-2, AAZC-4 and AAZC-6, respectively; the concrete not treated with NaOH is simply designated AAZC. Table 3 presents the basic parameters of these eco-friendly concrete samples.

The concrete batches were prepared in a concrete mixer, first adding the dry materials (aggregates, ZP, ASP and cement) and mixing them for 5 min. Subsequently, water was added and the combination was mixed for 5 min. Finally, NaOH and polycarboxylic acid water were incorporated and the composite slurry was mixed for 20 min. Each batch of concrete was poured into a cubical mould 10 cm on a side, and these specimens were used for all the subsequent tests and experiments.

The concrete specimens were wrapped in polyethene films and cured in a climatic chamber at T = 22 ± 1 °C and relative humidity >95% for 28 d. Specimens were removed for testing after being cured for 3, 7, 14 and, finally, 28 d. The plastic films were removed, and the specimens were left at room temperature (i.e., T = 22 ± 1 °C) and relative humidity = 50 ± 5% before testing.

### 2.3. Mechanical Tests

To evaluate the mechanical performance of the eco-friendly concrete specimens, their compressive strengths were measured on 100 mm × 100 mm × 100 mm specimens after 3, 7, 14 and 28 d of curing, according to the Chinese GB/T50081-2002 standard [21]. Three specimens per mixture were tested, and the average result was reported. After 28 d of curing, a split-tensile-strength test was also carried out on three cubic specimens (10 cm per side) according to the Chinese GB/T50081-2002 standard.

### 2.4. Microscopic Characterisation of AAZC, AAZC-2, AAZC-4 and AAZC-6

The samples after 28 d mechanical property tests of the above groups of concrete were selected to test the micro-mechanism of NaOH’s enhancement of the mechanical properties of eco-friendly concrete using SEM-EDS test, TG-DSC test, XRD test, FTIR test and NMR test. SEM (Tescan-Mira-Lms, Czech Republic) was employed to analyse the morphology of the carbon-coated concrete, and the chemical composition was determined using EDS and the Xplore 30 software. The hydration-product content of each concrete specimen was determined using a comprehensive synchronous thermal analyser over the temperature range of 30–1000 °C (TG–DSC, TA-SDT-Q600, TA Instruments, New Castle, DE, USA). In addition, XRD (Mini-Flex-600-X, Rigaku, Akishima, Japan) was used to determine the composition and crystalline structure of each concrete specimen with a scan range of 5–90°. The organic functional groups and the characteristic bands of the chemical-structure groups on the surfaces of the concrete specimens were determined using an FTIR spectrometer (Termo-Scientifict-IS-10, Walthman, MA, USA) in the range of 4000–400 cm^−1^. In addition, an NMR instrument (OMR Newmark MES-23-060V-I, Hangzhou, China) was used to measure the pore structure and content of each concrete specimen.

### 2.5. Freeze–Thaw Chloride-Penetration Experiment

The optimal grouping for AAZC excitation is derived from the mechanical property tests and micro-mechanism analysis tests above. The durability experiments were carried out with the optimal group and AAZC group concrete and OC group concrete, which led to the applicability of alkali-excited zeolite powder concrete with windlogged sand powder in salt-infested environments in cold and arid zones.

To simulate chloride penetration in a cyclical freeze–thaw environment, the concrete specimens were placed inside a custom-made freeze–thaw apparatus containing a 1.3 g/L NaCl solution. The experiment adopted the ‘fast-freezing method described in the ‘Test method for long-term performance and durability of ordinary concrete’ (GB/T50082-2009) [22]. The specimens were placed horizontally on cylindrical supports to ensure that the temperature was delivered from the surroundings, and they were exposed to 33 weekly freeze–thaw cycles (the lowest temperature was −16.15 °C, and the highest temperature was 2.85 °C). In brief, concrete specimens that had been cured for 24 d were soaked in a 1.3 g/L NaCl solution for 4 d at 16.85–22.85 °C, and the initial dynamic elastic modulus, mass and the compressive strength of each 100 mm × 100 mm× 100 mm specimen was measured after it had become saturated with the salt water. Finally, the mass of each specimen was measured, and the extent of mass loss, relative dynamic elastic modulus and loss of compressive strength were calculated after 25 freeze–thaw cycles. In addition, the experiment was stopped when any of the following three conditions were met: the number of freeze–thaw cycles reached 200, the mass loss exceeded 5%, or the elastic modulus fell below 60% of its initial value.

## 3. Results and Discussion

### 3.1. Compressive Strength and Tensile Splitting-Strength Experiment

**Figure 3:**
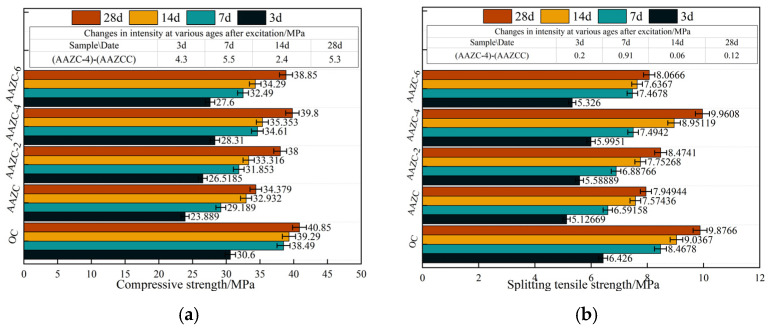
Mechanical properties of eco-friendly concrete before and after alkali treatment: (**a**) compressive strength and (**b**) split tensile strength.

Figure 3 shows the mechanical properties of the concrete specimens. As shown in Figure 3a,b, the compressive strength and split tensile strength of the concrete are lower than those of OC. According to relevant studies [23,24,25], the specific surface area of the ZP is larger than that of the cement; consequently, replacing cement with an equal mass of ZP increases the water requirement of the formulated concrete. This leads to a decrease in the water–cement ratio and a decrease in the amount of gel generated in the concrete, which decreases its mechanical properties. ASP has poor water absorption and less elemental Ca and Al [26]. Thus, a large dosage of ASP causes the content of C-S-H and C-A-S-H gels in the system to decrease dramatically. This increases the number of internal macropores and connecting pores, which in turn causes the mechanical properties of the concrete to decrease sharply. In addition, the compressive strength and split tensile strength of the AAZC concrete increased with increasing NaOH dosage, and both reached plateaus. Further, after being cured for 3, 7, 14 and 28 d, the compressive strengths of the AAZC-4 specimens increased by 22.6%, 27.5%, 19.8% and 17.2%, respectively, and the split tensile strengths increased by 21.3%, 20/6%, 18.2% and 16.3%. These mechanical properties of AAZC-4 specimens are similar to those of OC. This is similar to some studies [27] for NaOH treatment of the high territory. Their research showed that a NaOH treatment greatly improves the mechanical properties of concrete, prompted by the high territory. This occurs because NaOH accelerates the decomposition of the high territory so that it reforms into Na_2_SiO_3_ and NaAlO_2_ [28,29,30], and their reaction with the Ca(OH)_2_ produced by the hydration of the cement causes the formation of hydrated silicate and hydrated silica-aluminate gels, which makes AAZC-4 similar to OC. The gels fill the pore structures of the eco-friendly concrete, improving its compactness and strength [31].

Due to the lower addition of NaOH in AAZC-2, the ZP and ASP particles decompose insufficiently; thus, the effect of the volcanic ash on the composite cement material was improved but was lower than that of AAZC-4. In contrast, due to the greater addition of NaOH in AAZC-6, the additional NaOH forms a sol structure that wraps around the cement particles. This results in the cement not undergoing sufficient hydration reactions, thereby reducing the ability to improve the mechanical properties of this concrete specimen. Moreover, to explore the modification mechanism of eco-friendly concrete treated by NaOH, this study employed various characterisation techniques to analyse AAZC, AAZC-2, AAZC-4 and AAZC-6.

### 3.2. AAZC-4 Characterisation

#### 3.2.1. Surface Morphology and Elemental Content of AAZC-4

**Figure 4:**
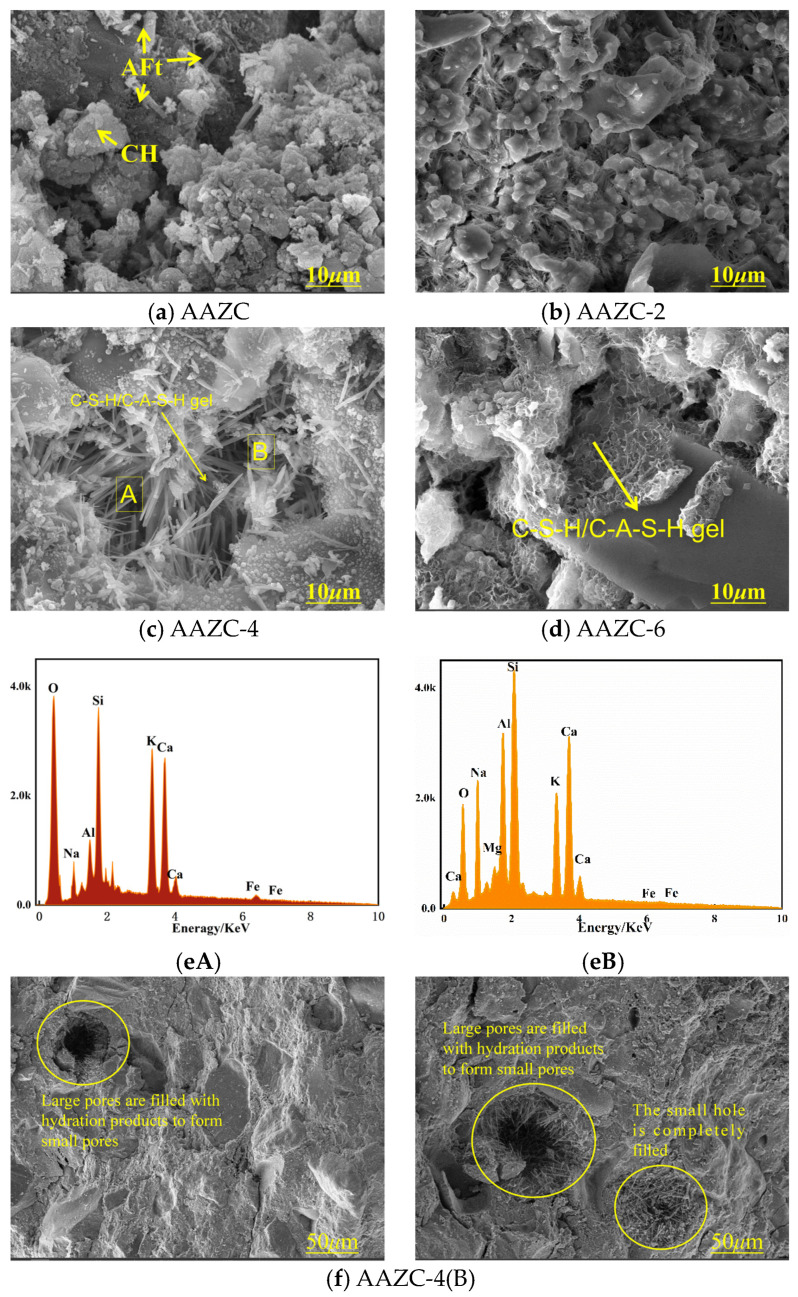
SEM images of the eco-friendly concrete specimens (**a**) AAZC, (**b**) AAZC-2, (**c**) AAZC-4 and (**d**) AAZC-6. (**e**) The EDS results for AAZC-4 ((**A**,**B**) are parallel samples). (**f**) Elongated prismatic hydrated-product crystals inside AAZC-4.

The surface morphologies of the constituent particles play a pivotal role in the mechanical properties of concrete, and the morphologies and EDS analyses of the prepared samples are shown in Figure 4. As shown in Figure 4a–d, the AAZC-2, AAZC-4, and AAZC-6 specimens exhibited fewer sheets of hydration products and more amorphous hydration products with fuzzy shapes than the untreated AAZC. However, AAZC-4 contained not only amorphous hydration products but also large amounts of elongated prismatic-crystal hydration substances. In addition, according to the EDS results shown in Figure 4e,f, AAZC-4 also contained elemental Ca, Si, Al, K, O and Ag.

#### 3.2.2. Thermogravimetric Analysis of AAZC, AAZC-2, AAZC-4 and AAZC-6

**Figure 5:**
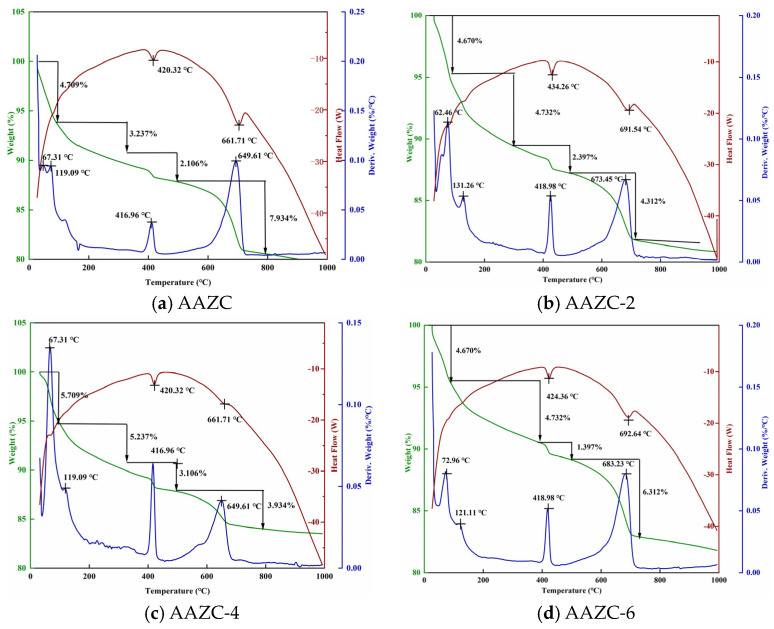
TG–DSC spectra of eco-friendly concrete specimens cured for 28 d.

The TG–DSC curves for AAZC, AAZC-2, AAZC-4 and AAZC-6 are presented in Figure 5. As the temperature increased from 298 to 493 °C, an endothermal peak appeared in all the eco-friendly concrete specimens. This demonstrated that the hydration products were losing the mass of free water and weakly bound water. Furthermore, the stable chemically bound water in the hydration products in AAZC, AAZC-2, AAZC-4 and AAZC-6 decomposed continuously between 493 and 873 °C, and destruction of the gel structure and Ca(OH)_2_ dehydration occurred between 873 and 1073 °C.

The untreated AAZC specimen exhibited higher mass loss (Figure 5c) than either AAZC-2 or AAZC-6 between 298 and 873 °C, and the mass loss increased by 1.039% when the temperature increased from 298 to 493 °C and by 2.224% between 493 and 873 °C. These results indicate that the NaOH treatment stimulated the generation of hydration products using the stable bound water inside the eco-friendly concrete when the amount of added NaOH was 4%. This also illustrated that NaOH, in moderation, promoted the decomposition of ZP and ASP and enabled the volcanic ash to have a better effect on the composite cement material. Thus, more stable hydration products are generated inside AAZC-4, and these products fill in the macropores, turning them into micropores, which improve their mechanical properties. This is also confirmed using the SEM observations.

#### 3.2.3. Phases and Functional Groups of AAZC-2, AAZC-4 and AAZC-6

**Figure 6:**
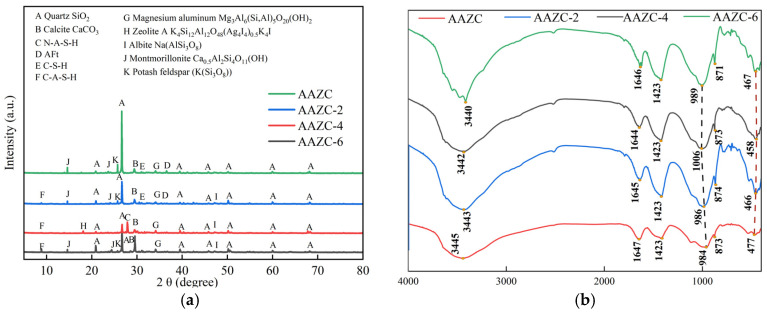
(**a**) XRD spectrum and (**b**) FTIR spectrum of eco-friendly concrete specimens before and after treatment.

As shown in Figure 6a, the hydration products of AAZC-2, AAZC-4 and AAZC-6 were different from those of the untreated AAZC. From the XRD spectrum, the untreated AAZC primarily contains quartz, mondestone, sodium length stone and the C-A-S-H gel, which is consistent with previous research. However, when NaOH was added to the eco-friendly concrete, the characteristic peak corresponding to mondestone disappeared, the detected peaks—which corresponded to sodium length stone and quartz—became weak, and the characteristic peaks due to the C-S-H and C-A-S-H gels appeared. These results demonstrate that the addition of NaOH changed both the original hydration process and its products. Moreover, AAZC-4 had an obvious diffraction peak due to A-type potassium zeolite crystals, and the characteristic diffraction peak (zeolite precursor) at 2θ = 29° matched well with the N-A-S-H gel from AAZC-4. Therefore, the reasons for the notable discrepancies between the properties of the eco-friendly concrete specimens with added NaOH (AAZC-2, AAZC-4 and AAZC-6) and those of untreated AAZC were that the existing OH^−^ and the OH^−^ from the decomposed composite material reacted with Ca(OH)_2_ and generated C-S-H, C-A-S-H, N-A-S-H and A-type potassium zeolite crystals [32,33,34]. Figure 6b also shows the FTIR spectra of these four concrete specimens. The broad band at 900–1300 cm^−1^ was due to hydration products and was mainly caused by the asymmetric vibrations of the Si–O–T structure (where T = Si or Al) in the gel products.

**Figure 7:**
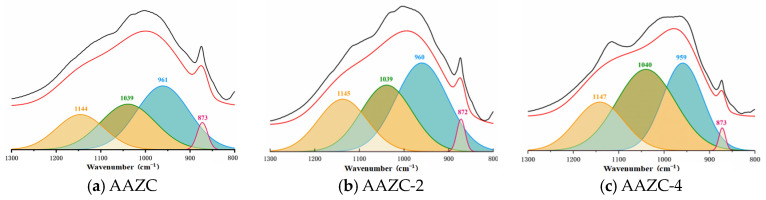

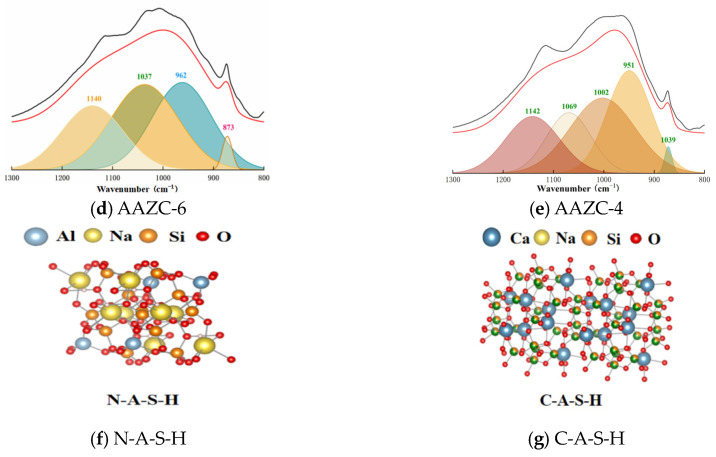
FTIR split diagram and Crystal diagrams.

Figure 7 presents the elemental analysis obtained from the FTIR spectra. The Si-O vibrations from SiO_4_ were detected in AAZC-2 (466 cm^−1^), AAZC-4 (458 cm^−1^), AAZC-5 (477 cm^−1^) and AAZC-6 (467 cm^−1^), indicating that the SiO_2_ had partially dissolved. The degree of polymerisation of SiO_2_ is thus reduced, and silicate and aluminosilicate gels are generated. The new Si-O-T peak exhibits little difference between AAZC-2, AAZC-6 and untreated AAZC, demonstrating that they contain similar hydration products. However, the shift of the Si-O-T stretching vibrations from 984 to 1006 cm^−1^ when the added NaOH was increased from 0% to 4% demonstrates the high degree of polymerisation of the aluminosilicate gel products generated inside the AAZC-4 specimen. Furthermore, the broadband at 800–1300 cm^−1^ corresponded to primary absorption peaks; the broad bands detected at 800–900, 900–980, 980–105 and 1050–1300 cm^−1^ were associated with AAZC-2, AAZC-4, AAZC-6 and AAZC, respectively. According to Figure 7a–d, the characteristic broadband diffraction peak at 800–900 cm^−1^ corresponds to nonpolymeric silicate. The area under its deconvolution peak decreased as the amount of NaOH increased, but the deconvolution peak areas of calcium silicate hydrate (900–980 cm^−1^) and hydrated silico-aluminate (980–1050 cm^−1^) rapidly increased. At the same time, the relative concentration of Si-O-T, which appeared in the range 980–1050 cm^−1^, increased from 32.67% to 42.76%, indicating that a new crystal substance had been generated. Two strong broadband features were also detected at 1002–1050 cm^−1^ from AAZC-4; they are due to N-A-S-H, and they indicate that a large amount of the N-A-S-H gel with a high degree of polymerisation had been generated. Its structure is presented in Figure 7f. Due to the hydration of the cement, numerous Ca^2+^ replaced the Na^+^ from the N-A-S-H gel to generate a C-A-S-H gel with a low degree of polymerisation (Figure 7g). Thus, combined with the SEM and TG–DSC results, these FTIR results show that when the added NaOH was 4%, the eco-friendly concrete (AAZC-4) was characterised by high mechanical properties due to the increased amounts of the C-A-S-H gel, the C-S-H gel, the N-A-S-H gel and A-type potassium zeolite crystals.

#### 3.2.4. NMR Study of AAZC-2, AAZC-4 and AAZC-6

From the characterisation results discussed above, AAZC-4 contained more hydration products than any of the other concrete specimens. This section describes the results of the NMR studies we used to analyse and compare the pore structures of the eco-friendly concrete specimens, which revealed the mechanism responsible for improving their mechanical properties.

**Figure 8:**
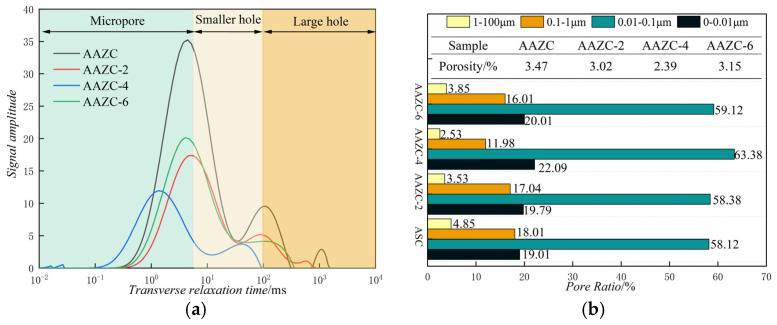
(**a**) *T*_2_ spectrum and (**b**) pore proportions of eco-friendly concrete specimens before and after alkali treatment.

Figure 8 shows the spectra and pore proportions of the eco-friendly concrete specimens before and after treatment with NaOH. The area of *T_2_* indirectly represents the quantity of pores in the concrete. The transverse relaxation time corresponds to the pore size; the shorter the transverse relaxation time, the smaller the pore size. As shown in Figure 8a, the *T*_2_ spectrum can be divided into three spectral intervals, which represent micropores, mesopores and macropores, respectively, in the AAZC, AAZC-2, AAZC-4 and AAZC-6 specimens after being cured for 28 d. Furthermore, compared with untreated AAZC, the areas under the three other spectra obviously decreased. In addition, the *T_2_* spectrum shifted in AAZC-4 but not in either AAZC-2 or AAZC-6. These results demonstrate that for AAZC-4, the quantity of pores decreased and the density increased, which improved its mechanical properties.

It has been reported that the pore diameters of concrete can be divided into four grades: 0.00–0.01, 0.01–0.10, 0.10–1.00, and 1.00–100.00 μm [35,36]. They have been classified as harmless pores, little-damage pores, harmful pores and multi-damage pores, respectively. Generally, the proportion of small pores (including harmless pores and little-damage pores) plays a notable role in the mechanical properties of concrete. Thus, an analysis of the pore distributions and proportions in eco-friendly concrete specimens with different NaOH additions was performed. According to the results presented in Figure 8b, in AAZC-2 and AAZC-6, the total proportion of harmful and multi-damage pores decreased by 0.12% and 0.10%, respectively, compared with those of AAZC, while the proportion of harmless and little-damage pores increased by 0.06% and 0.04%, respectively, and the overall porosities decreased by 0.03% and 0.08%, respectively. These results demonstrate that the overall porosity of the concrete specimens with 2% and 6% added NaOH had little effect on the improvement in the mechanical properties. In comparison, for AAZC-4, the proportion of harmful pores and multi-damage pores decreased by 0.43%, while the proportion of harmless pores and small-damage pores increased by 0.21%, and the total pore proportion decreased by 0.22%. In addition, the decreased proportion of harmful pores and multi-damage pores was converted into 0.21% of harmless pores and little-damage pores, and the remaining harmful pores and multi-damage pores were filled. Therefore, these results demonstrate that when the amount of NaOH added was 4%, the macropores were filled with the C-S-H gel, the C-A-S-H gel, the N-A-S-H gel and A-type potassium zeolite crystals in this eco-friendly concrete specimen, which increased the density of the concrete and enhanced its mechanical properties. These results were confirmed using the SEM, XRD and FTIR experiments.

### 3.3. Mechanism of NaOH Lifting for AAZC

**Figure 9:**
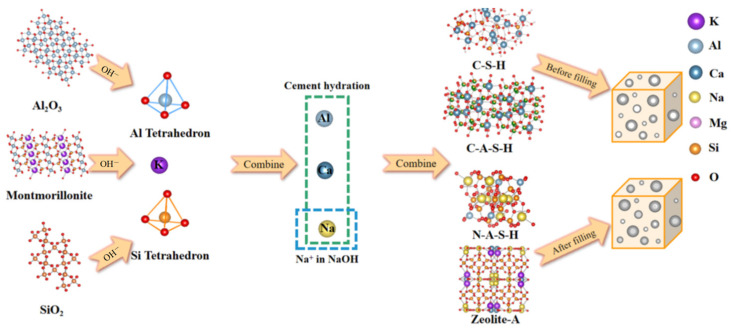
Mechanical properties of AAZC-4 before and after treatment with NaOH, illustrating the enhancement mechanism.

As mentioned above, AAZC-4 has been identified as the optimal eco-friendly concrete. Coupled with the discussions in Section 3.2, a general mechanism for enhancing the mechanical properties of AAZC-4 is proposed here and presented in Figure 9. Montmorillonite, alumina and silica in zeolite powder and airborne sand powder generate silicate and meta-aluminate under the excitation of sodium hydroxide and then react with calcium hydroxide generated using cement hydration to produce C-S-H gel, C-A-S-H gel, which initially fills the pore space, and then the reaction further produces N-A-S-H gel and potassium A zeolite, which further fills the pore space. According to published studies, following the theory of alkali excitation. The results show that the main mechanism responsible for the enhancement of AAZC-4 was the generation of new hydration products in both macropores and micropores. Under the impact of OH^−^ from NaOH, the SiO_2_, Al_2_O_3_ and montmorillonite are converted into silica and aluminium tetrahedrons in the eco-friendly concrete. Next, the new substances are combined with elemental Ca and Al and are hydrated by the cement to produce more beneficial gels and higher-strength A-type potassium zeolite crystals. Finally, the gels and A-type potassium zeolite crystals combined to fill the pores in AAZC-4, improving its internal microstructure remarkably and enhancing its mechanical properties.

### 3.4. Freeze–Thaw Chloride-Penetration Experiment

**Figure 10:**
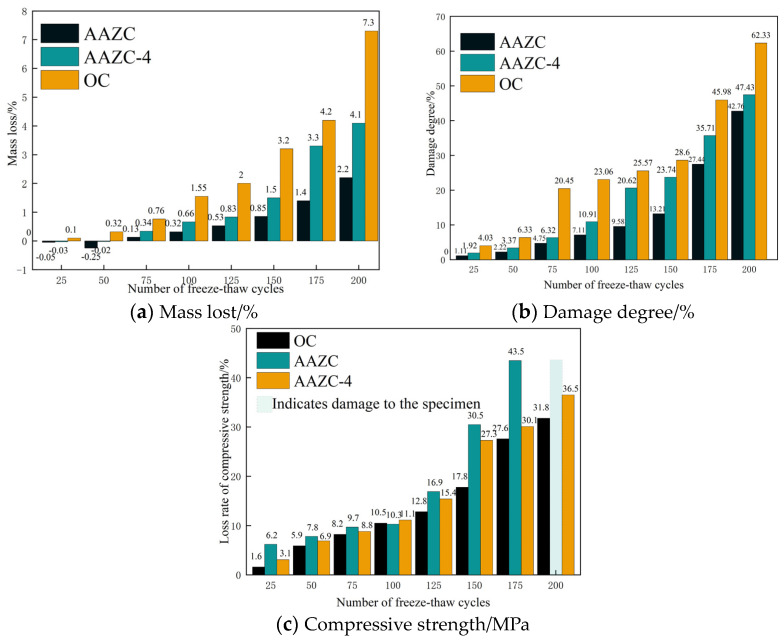
(**a**) Mass loss, (**b**) degree of damage and (**c**) compressive strength of AAZC-4 as functions of the number of freeze–thaw cycles.

Figure 10a–c shows the extent of mass loss, degree of damage and loss of compressive strength of OC, AAZC and AAZC-4 in a freeze–thaw chloride-penetration environment with 1.3-g/L NaCl [37,38]. Here, the mass loss represents the degree of erosion of the concrete during the freeze–thaw cycles, and the degree of damage represents the overall change in the density of the concrete during those cycles. The parameter can be calculated from the elastic modulus. Both the extent of mass loss and the degree of damage increased gradually with the number of cycles under different working conditions for OC, AAZC and AAZC-4, indicating that both internal and external structures of the concrete were destroyed in this test. In addition, the mass loss and degree of damage of OC, AAZC and AAZC-4 were approximately constant before 150 cycles, but each concrete specimen experienced accelerated erosion and rapidly increasing damage after 150 cycles. This demonstrates that cycle 150 marks a critical point for the mass loss and degree of damage for all of these concrete specimens. In addition, the mass loss, degree of damage and compressive strength of OC and AAZC-4 remained relatively close to each other even after 200 cycles. However, these properties of AAZC-4 were substantially different from those of untreated AAZC after 200 cycles; the extent of mass loss, degree of damage and compressive strength of AAZC were 4.3%, 51% and 35.2%, respectively, whereas those of AAZC-4 were 6.6%, 63% and 13.6%, respectively, demonstrating that the untreated AAZC cannot satisfy normal working requirements under these conditions. The results also demonstrated that compared with untreated AAZC, the mass loss of AAZC-4 decreased by 36.5%, the degree of damage was reduced by 19%, and the compressive strength decreased by 52.1%. Thus, all the results indicate that AAZC-4 had characteristics similar to those of OC while undergoing freeze–thaw chloride penetration and that it exhibited sustainable working performance in the composite chloride-penetration environment.

## 4. Conclusions

Herein, a novel alkali-treated eco-friendly concrete (AAZC-4) was prepared by adding ZP, ASP and NaOH and decreasing the corresponding amount of cement. The mechanical properties of the resulting concrete specimen were evaluated, and the mechanism responsible for enhancing its mechanical properties was revealed in this study. The following conclusions were drawn:

1. With m(ZP):m(ASP) = 5:5 and the amount of added NaOH fixed at 4%, the most favourable eco-friendly concrete (AAZC-4) was obtained with the best mechanical properties among the alkali-treated AAZC specimens. Compared with untreated AAZC, the compressive strength of AAZC-4 increased by 22.6%, 27.5%, 19.8% and 17.2%, whereas the split tensile strength increased by 21.3%, 20.6%, 18.2% and 16.3% after curing for 3, 7, 14 and 28 d, respectively.
2. According to SEM–EDS results, the surface of AAZC-4 produced a series of hydration products similar to those of untreated AAZC, as well as special elongated prismatic hydration products that included Ca, Si, Al, Na, K, O and Mg.
3. AAZC-4 contained more stable hydration products—such as the C-S-H, C-A-S-H and N-A-S-H gels—as well as elongated prismatic A-type potassium zeolite crystals than did the other alkali-treated AAZC specimens.
4. For AAZC-4, the area of *T_2_* considerably decreased, and the deviation to the left was greater than for any of the other concrete specimens. The total porosity of AAZC-4 also decreased, with a decrease of 0.43% in the number of macropores and an increase of 0.21% in the number of harmless pores and little-damage pores. This increased the density of AAZC-4 and enhanced its mechanical properties.
5. Compared with untreated AAZC, the mass loss, degree of damage and loss of compressive strength of AAZC-4 were reduced by 36.8%, 19% and 52.1%, respectively, after 200 cycles in a composite chloride-penetration environment. These characteristics of AAZC-4 while undergoing freeze–thaw cycles are similar to those of OC. Thus, the AAZC-4 specimen had sustainable working performance in the chloride ion permeable environment in cold and arid areas.

## Figures and Tables

**Table 1:** Cement characteristics.

Chemical Composition (%)							Physical–Mechanical Properties					
SiO_2_	Al_2_O_3_	CaO	Fe_2_O_3_	MgO	SO_3_	Other	Setting Time (min)		Compressive Strength (MPa)		Flexural Strength (MPa)	
							Initial	Final	3 d	28 d	3 d	28 d
21.42	5.43	63.64	3.04	2.82	2.17	1.69	180	395	24.8	48.9	5.0	8.1

**Table 2:** Chemical composition and particle characteristics of wind-blown sand and zeolite powders.

Powder	Chemical Composition (%)						Particle Characteristics			
	SiO_2_	Al_2_O_3_	CaO	Fe_2_O_3_	K_2_O	Other	Volume Mean Diameter (μm)	Specific Surface Area (m^2^/kg)	D50 (μm)	D98 (μm)
ASP	68.5	12.1	7.31	4.4	2.51	5.18	13.56	994.72	7.26	67.97
ZP	73.4	11.91	2.16	1.86	3.14	7.53	20.44	594.91	13.19	91.16

**Table 3:** Parameters of the eco-friendly concrete samples.

Sample	Cement	ZP	ASP	River Sand	NaOH Activator	Coarse Aggregate	Water	Additives
	(kg/m^3^)	(kg/m^3^)	(kg/m^3^)	(kg/m^3^)	(kg/m^3^)	(kg/m^3^)	(kg/m^3^)	(kg/m^3^)
OC	489.8	0.0	0.0	761.9	0	1777.7	210.6	3.3
AAZC	244.4	122.2	122.2	761.9	0	1777.7	210.6	3.3
AAZC-2	244.4	122.2	122.2	761.9	4.88	1777.7	210.6	3.3
AAZC-4	244.4	122.2	122.2	761.9	9.78	1777.7	210.6	3.3
AAZC-6	244.4	122.2	122.2	761.9	14.66	1777.7	210.6	3.3

## Data Availability

Data are contained within the article.
